# The splicing fate of plant SPO11 genes

**DOI:** 10.3389/fpls.2014.00214

**Published:** 2014-05-21

**Authors:** Thorben Sprink, Frank Hartung

**Affiliations:** Biosafety in Plant Biotechnology, Julius Kühn InstituteQuedlinburg, Germany

**Keywords:** SPO11, *Arabidopsis thaliana*, alternative splicing, meiosis, double strand breaks

## Abstract

Toward the global understanding of plant meiosis, it seems to be essential to decipher why all as yet sequenced plants need or at least encode for two different meiotic *SPO11* genes. This is in contrast to mammals and fungi, where only one SPO11 is present. Both SPO11 in *Arabidopsis thaliana* are essential for the initiation of double strand breaks (DSBs) during the meiotic prophase. In nearly all eukaryotic organisms DSB induction during prophase I by SPO11 leads to meiotic DSB repair, thereby ensuring the formation of a necessary number of crossovers (CO) as physical connections between the homologous chromosomes. We aim to investigate the specific functions and evolution of both *SPO11* genes in land plants. Therefore, we identified and cloned the respective orthologous genes from *Brassica rapa*, *Carica papaya*, *Oryza sativa*, and *Physcomitrella patens.* In parallel we determined the full length cDNA sequences of SPO11-1 and -2 from all of these plants by RT-PCR. During these experiments we observed that the analyzed plants exhibit a pattern of alternative splicing products of both SPO11 mRNAs. Such an aberrant splicing has previously been described for Arabidopsis and therefore seems to be conserved throughout evolution. Most of the splicing forms of *SPO11-1* and *-2* seem to be non-functional as they either showed intron retention (IR) or shortened exons. However, the positional distribution and number of alternative splicing events vary strongly between the different plants. The cDNAs showed in most cases premature termination codons (PTCs) due to frameshift. Nevertheless, in some cases we found alternatively spliced but functional cDNAs. These findings let us suggest that alternative splicing of SPO11 depends on the respective gene sequence and on the plant species. Therefore, this conserved mechanism might play a role concerning regulation of SPO11.

## Introduction

In most eukaryotic organisms the rearrangement of the parental alleles by homologous recombination during meiosis is one essential step leading to genetic diversity. Correct pairing and subsequent homologous recombination in prophase I ensure stability of the chromosome number on the one hand and variability in the developing cells due to crossover resolution resulting in exchange of genetic material between the homologous chromosomes on the other hand. One crucial aspect in the arrangement of the recombination progress is the initial formation of double strand breaks (DSBs) by SPO11. The eukaryotic SPO11, which shows homology to the archaeal Topoisomerase VIA subunit (TOPVIA), is one of the key factors mediating the formation of DSBs in a wide range of organisms (Bergerat et al., 1997; Keeney et al., 1997; Grelon et al., 2001). Without DSBs and their subsequent repair as crossovers there is no physical linkage between the homologous chromosomes and random chromosome distribution would appear (Cole et al., 2010). Like TOPVIA, SPO11 is able to cleave DNA via a 5′ phosphotyrosyl linkage thereby defining the acceptor sites of exchange between the parental genomes (Cole et al., 2010). In contrast to animals and fungi where a single SPO11 is sufficient for meiotic DSB formation, plants encode for at least two SPO11, referred to as SPO11-1 and -2, that are both essential in a functional protein form for DSB formation during meiosis (Keeney et al., 1997; Grelon et al., 2001; Hartung et al., 2007; Shingu et al., 2012). However, the mechanism by which two very different SPO11 proteins in plants induce DSBs specifically during meiosis is still unclear. Our long term aim is to investigate the specific functions, origin and evolution of each SPO11 in the plant kingdom. By analyzing complete genomic sequences of more than 40 plants, we were able to show that all as yet sequenced land plants encode for at least three *SPO11* genes. Two of them, AthSPO11-1 and -2 play a meiotic role. The third one, AthSPO11-3 together with TOPVIB, the second subunit of the topoisomerase, possesses essential functions during somatic development of plant cells but plays no role in meiosis (Hartung et al., 2002a, 2007; Stacey et al., 2006; Simkova et al., 2012).

The phylogenetic analyses of SPO11-1 and -2 in land plants and algae show very clearly that both genes are highly conserved and ancient in the lineage of plants but cannot be found in algae or protists in the same form. An analysis of a high number of available genomic and protein sequences of *SPO11* in virtually all kingdoms of life shows that at least one duplication of the original *SPO11* from archae must have occurred very early preceding the split of animals and plants (Malik et al., 2007; this work). In addition to this, the intron content and localization in the *SPO11* genes from different organisms shows ancestral conservation between animals, fungi, and plants but also dramatic variations in protists and green algae (Hartung et al., 2002b; this work).

Early investigations of SPO11-1 expression in *Arabidopsis thaliana* exhibited an extensive pattern of alternative splicing, which we were now able to show also for SPO11-2 (Hartung and Puchta, 2000). Analyzing the expression in other plants we could identify various non-functional alternative splicing events for *SPO11-1* and *-2* in *Oryza sativa*, *Brassica rapa*, *Carica papaya*, and *Physcomitrella patens*. Additionally, we found putative functional forms of alternative spliced *SPO11-1* or *-2* for the first time in plants, namely in *B. rapa*, *C. papaya*, *O. sativa*, and *P. patens*. The fact that both SPO11 show such a diversified splicing pattern and that alternative splicing for both SPO11 is conserved between the different species indicates that SPO11 has an ancient complex transcriptional regulation mechanism, most probably involving the non-sense mediated decay pathway as described for other meiotic genes (Chiba and Green, 2009).

## Materials and methods

### Accession numbers

We sequenced the cDNA of *SPO11-1* and *SPO11-2* from *B. rapa*, *C. papaya*, and *P. patens*. The resulting sequences have been deposited in this order in the NCBI database under accession numbers KF841348, KF841349, KF841350, KF926859, KF926860, and KF926861.

### Plant material and growth conditions

Arabidopsis (*Arabidopsis thaliana L.*) wild type plants (Col-0) were seeded on a 3:1 mixture of soil and vermiculite spiked with 4 g/l Plantacote (Wilhelm Haug GmbH und Co. KG, Ammerbuch, Germany) as fertilizer and 0, 4 g/l Exemptor (BAYER crop science, Langenfeld, Germany) as an preventive insecticide. Plants were kept under short day conditions (8-h light/16-h dark cycle at 18°C) for 3 weeks and then transferred to a green house under a long day regime (16-h light/8 h- dark at 20°C). Rice (*O. sativa subsp. Japonica*) plants were grown in the greenhouse under a long day regime as well as *B. rapa* var. fastplant. Papaya (*C. papaya L.*) trees were grown in a public tropical greenhouse on loamy soil. *P. patens* gametophores were kindly provided by Gertrud Wiedemann from the group of Ralf Reski (Freiburg, Germany) on solid media.

### Gene compilation and source of sequence data

A total of 42 *SPO11-1* and 39 *SPO11-2* sequences from land plants were extracted from different databases using the Arabidopsis and *O. sativa* orthologs as starting point. The databases used were: Phytozome (http://www.phytozome.net), JGI (http://www.jgi.doe.gov), Ensembl plants (http://plants.ensembl.org/index.html), Gramene (http://www.gramene.org/), CoGeBlast (http://genomevolution.org/r/5kv5), and NCBI (http://www.ncbi.nlm.nih.gov/genomes/PLANTS/PlantList.html). Models predicting not the full length cDNA but only a few assembled ESTs were manually curated by aligning these sequences to annotated *SPO11-1* and *-2* of *A. thaliana* as well as *O. sativa* using MegAlign (DNASTAR Inc. Madison, WI, USA). For some species the ESTs and the cDNA prediction did not cover the whole sequence. In these cases, the corresponding genomic DNA region was screened for possible matches and manually added to the model if possible. To check the accuracy of our prediction, elected coding sequences (CDS) were amplified using Primers covering the whole predicted CDS (Supplemental Table 1). The sequence of each gene was checked by sequencing, using the Sanger method (GATC Biotech AG, Konstanz, Germany). All sequences used for phylogenetic comparisons and their accession codes are listed in Supplemental Tables 2, 3.

### RNA isolation and used tissue

All kits used in this study were used following the instructions of the manufacturer. Total RNA was isolated using the Bio & Sell RNA mini Kit (Bio&Sell e.K., Feucht, Germany). To evaluate the abundance of SPO11 transcripts in generative tissue, fresh young flowers were used for RNA isolation. In the case of *C. papaya*, flowers were stored in RNAshield (Zymo research Europe GmbH, Freiburg, Germany) prior to RNA isolation. To check the abundance in vegetative tissue, leaf material was used. In the case of *C. papaya* no leaf material was available so fruit exocarp tissue was utilized instead. To check expression in *P. patens* 6-week old gametophores were used for RNA Isolation. Isolated RNA was treated with DNase I (Thermo Fisher Scientific, Germany). To check contamination with genomic DNA in the treated RNA, a PCR was performed with RNA as a template. No contamination was found in the RNA samples after DNase treatment (data not shown). cDNA was produced using an anchored oligo dT Primer with the Maxima H Minus Reverse Transcriptase Kit (Thermo Fisher Scientific, Germany) using 2–4 μg of total RNA as a template for the RT-reaction.

### Molecular characterization of SPO11

Reverse transcribed cDNA was used as a template for a PCR reaction using 50 amplification cycles. The resulting PCR products were purified using the GeneJET PCR purification Kit (Thermo Fischer Scientific, Germany) and cloned into the insTA-cloning vector system (Thermo Fischer Scientific, Germany). Resulting clones were screened in a colony PCR using M13 Primer. Clones differing in the size of their insert were sequenced and analyzed using MegAlign.

## Results

### Identification of SPO11 homologous among the plant kingdom

The progress in sequencing and the growing amount of data input into the sequence databases provided us with a powerful tool for the identification of putative homologous proteins in a rapidly growing number of organisms by database searches using common bioinformatics tools such as BLAST-programs (TBLASTN = protein sequence search against the respective genomic sequence). By using known sequences of SPO11 from *A. thaliana* and *O. sativa* we were able to identify orthologs to SPO11-1 and -2 in all publicly available land plant genome assemblies sequenced to date. The identities of the orthologs to SPO11-1 from *A. thaliana* ranges between 95.9% for *Arabidopsis lyrata* to 45.4% for *P. patens*. The identities of the orthologs to SPO11-2 from *A. thaliana* is comparable to the identities found for SPO11-1. For *A. lyrata* the identity is 96.9% and the least identity is found again for *P. patens* with 47.5% (Supplemental Tables 2, 3). In both cases, the monocotyledonous plants show approximately 10% less identity compared to the dicotyledonous plants representing the earlier split of mono- and dicots (Supplemental Tables 2, 3).

In our database analyses we found orthologs of SPO11-1 and -2 in all land plants with completely sequenced genomes. The conserved gene structure of *SPO11-1* in land plants contains 15 exons and 14 introns in the coding region. This structure has been verified earlier by sequencing of the cDNAs from *A. thaliana* and *O. sativa* (Hartung and Puchta, 2000; Jain et al., 2006). In a large number of cases, the annotation of these orthologs corresponded to the known cDNAs but in several cases the correspondence was incomplete. In virtually all of the latter cases we could perform a manual correction according to the known sequences. In the Asterid *Utricularia gibba* we found that intron number one was missing, clearly indicating an intron loss event in this species. In Table 1 the predicted position and phase of the introns in relation to their deduced protein sequence is given. All plants with a completely sequenced genome possess *SPO11-2* and show a conserved gene structure concerning the position of the 10 introns in the coding region of *SPO11-2* (Table 1). However, we can identify three exceptions. Firstly, *Malus domestica, Prunus persica, Vitis viniferis, Fragaria vesca*, and *Eucalyptus grandis* all miss the first intron so it has most probably been lost in a common ancestor of these species. Secondly, in some rice species a loss event of intron two occurred, as this intron is missing only in *O. sativa* and *O. glaberrima*. This intron loss event must have occurred recently as the close relative *O. brachyantha* contains intron two. Thirdly, the plant *Aquilegia coerulea*, belonging to the *Ranunculaceae*, encodes for a *SPO11-2* gene which does not contain a single intron (Supplemental Figure 1). Most probably this *SPO11-2* gene is a reinserted copy of a fully spliced reverse transcribed mRNA, a mechanism which is also proposed to have resulted in the origin of *SPO11-3* (Hartung et al., 2002b).

**Table 1:**
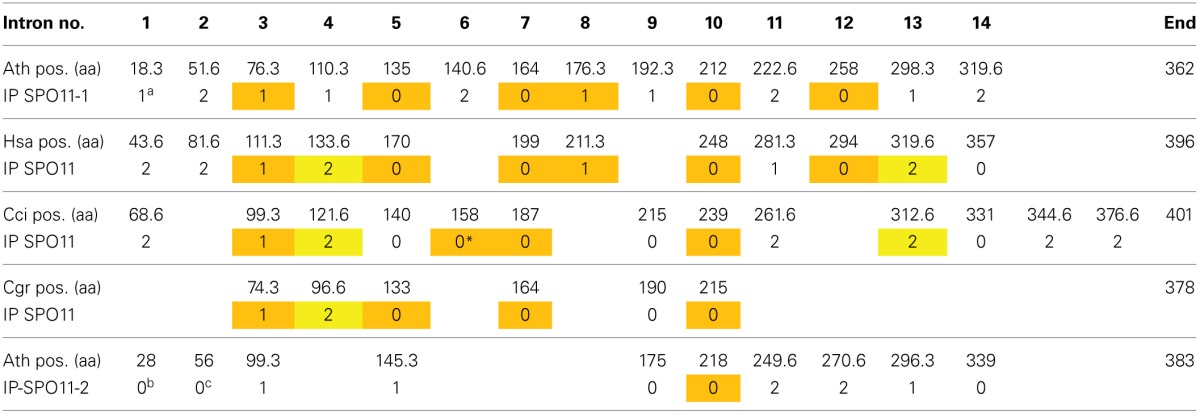
**Intron localization of *A. thaliana*, *H. sapiens*, and the SPO11 genes from the two fungi *C. cinerea* (Basidiomycota) and *C. grayi* (Ascomycota) with respect to their corresponding amino acid sequence positions**.

The numbering of introns was done with respect to the highest number of 14 introns in Arabidopsis SPO11-1. Gaps are included in the other lines to better visualize the conserved intron positions.

^a^This intron has been lost in Utricularia gibba.

^b^This intron has been lost in Fragaria vesca, Malus domestica, Mimulus guttatus, Prunus persica, and Vitis vinifera.

^c^This intron has been lost in Oryza brachyantha and Oryza sativa.

^*^This intron number 5 of C. cinerea is in the same conserved position as intron number 5 of Arabidopsis SPO11-1 and H. sapiens but is preceded by a non-conserved intron position (no. 4).

Color coding: Orange, intron position conserved at least since the split of the plant and animal kingdom, sometimes (8 and 12) lost later on in fungis; Yellow, intron position conserved between H. sapiens (as representative for animals) and two fungal divisions. Abbreviations: IP, Intron position; Ath, Arabidopsis thaliana; Cci, Coprinopsis cinerea; Cgr, Cladonia grayi; Hsa, Homo sapiens.

Considering all this, it is very clear that SPO11-2 existed before the evolution of land plants that took place approximately 450 mya, exemplarily shown by the *SPO11-2* sequence (genomic and cDNA) of the moss *P. patens*, an extant member of one of the oldest land plant lines (Supplemental Figure 1). However, there is a recognizable gap of conservation considering a second or third *SPO11* gene in green algae and other algae that belong to the heterokontophyta or rhodophyta. All fully sequenced green algae contain a single *SPO11* gene that shows the highest sequence identity to SPO11-3 from land plants. In all of these algae, the second subunit TOPVIB is also present as has been shown earlier by Malik et al. (2007). This indicates that like land plants, algae most probably possess a functional complex of TOPVIA and B. A very interesting feature of the *SPO11-3* gene structure in green and other algae is that this gene possesses a high number of introns (14 in *Chlamydomonas reinhardtii*) that are not correlated to the introns found in plant *SPO11-1* or *-2*, whereas *SPO11-3* in land plants possesses only one intron (whose position is corresponding to intron no. 6 of *CreSPO11-3*) or none at all (Supplemental Figure 2).

Malik et al. (2007) performed extensive phylogenetic analyses in which they described a second *SPO11* gene that can be found in chlorophyta (prasinophyceae), rhodophyta, and heterokontophyta and is by its sequence homology most related to *SPO11-2* of plants. However, a meiotic function of the gene has not been demonstrated for any of these organisms so far, and additionally, the gene structure is highly different compared to *SPO11-2* from land plants (Supplemental Figure 2). The *SPO11-2* similar genes of phylogenetically very different algae either possess no intron at all, or a much smaller number of introns in positions that are not correlated to the highly conserved positions found in all land plant *SPO11-2* orthologs (Supplemental Figure 2). Taking all data together, two very early duplications of the original *SPO11-3* (which is orthologous to *TOP6A* from archea) must have occurred, followed by a number of losses in different kingdoms.

This raises the question if SPO11-2 from algae is really orthologous to SPO11-2 from land plants. To address this question, we can use the method of comparison of intron positions which we already developed earlier (Hartung et al., 2002b). In brief, after the alignment of the protein sequences, each intron position is projected onto these sequences which can result in an intron located in between two coding triplets (phase 0) or interrupting a coding triplet after the first or second nucleotide (phase 1 and 2 which results in e.g., amino acid 18.3 or 18.6, respectively). Doing so for all genes, we can clearly see that six intron positions in *SPO11-1* are conserved throughout the animal and plant kingdom, spanning a time frame of almost one billion years (Table 1; Hartung et al., 2002b). These introns are number 3, 5, 7, 8, 10, and 12 with respect to the *AthSPO11-1* gene (Table 1). The ancient intron positions 8 and 12 most probably have been lost after the divergence of plants and animals/fungis in the fungi kingdom only. Furthermore, even one intron of *SPO11-2* (no. 6) is somehow conserved with respect to fungal *SPO11* which is a single copy *SPO11* (Hartung et al., 2002b). These conserved intron positions cannot be found in the second *SPO11* copy in algae or protists (Supplemental Figure 2). Considering this, we think that the second *SPO11* in protists and algae is an ortholog of plant *SPO11-2* due to its sequence conservation but a lot of changes concerning its gene structure have taken place during evolution (Malik et al., 2007; this work).

### Analysis of SPO11 cDNAs

**Figure 1:**
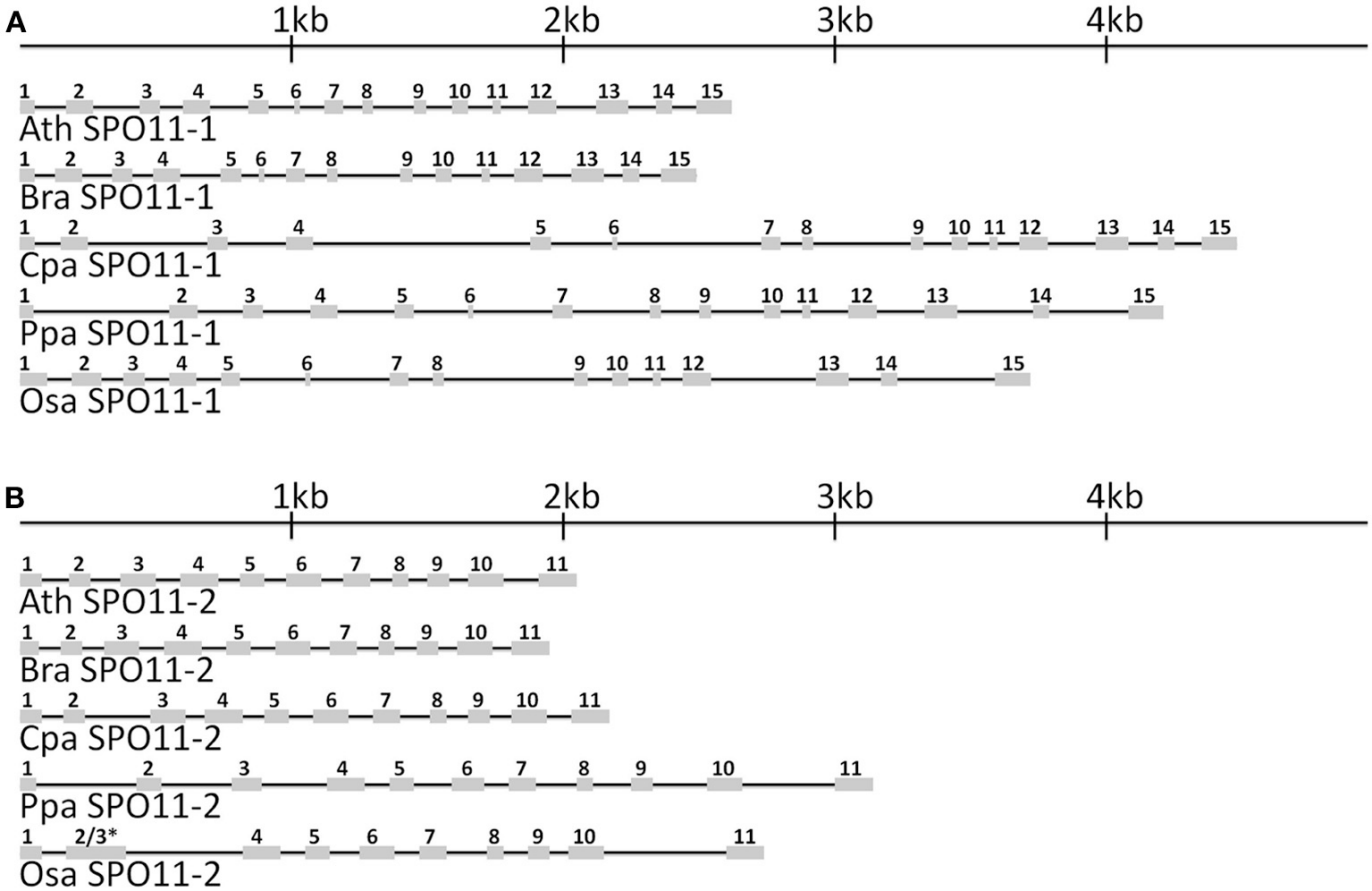
**The in-scale exon–intron organization of SPO11-1 (A) and SPO11-2 (B) for five analyzed species**. Ath, *Arabidopsis thaliana*; Bra, *Brassica rapa*; Cpa, *Carica papaya*; Ppa, *Physcomitrella patens*; Osa, *Oryza sativa*. Coding regions are represented by gray boxes. The introns are represented by black lines. ^*^ Intron 2 has been lost in OsaSPO11-2. For a better comparison exon 2 was marked with 2 and 3 due to their fusion.

Based on the obtained database sequences, we designed primer pairs to amplify the whole coding sequence (CDS) of *SPO11-1* and *SPO11-2* from *B. rapa, C. papaya, O. sativa*, and *P. patens*. The predicted models fit the amplified CDSs in all cases. Using preamplified cDNA of the corresponding species, both *SPO11* could be amplified in their full length from *C. papaya*, *B. rapa*, and *A. thaliana*. From *P. patens* and *O. sativa* only *SPO11-1* could be amplified as a full length construct, for *SPO11-2* from *P. patens two* overlapping fragments were amplified, sequenced, and artificially put together afterwards. For *O. sativa* no full length construct of *SPO11-2* could be amplified due to high GC content in the 5′ region (GC > 80%). Every time we tried to evaluate *SPO11-2* all constructs were artificially modified due to a repetitive sequence in the 5′ region. Due to this artificial error *SPO11-2* from *O. sativa* was not further analyzed in detail. In this region the PCR leaped directly from one repetitive sequence to the next, resulting in constructs without a methionine that could not possibly be spliced in a natural way. The structures of the *SPO11-1* and *-2* genes are shown schematically in Figure 1. In all cases, *SPO11-1* consists of 15 exons and 14 introns. *SPO11-2* codes for 11 exons interrupted by 10 introns in all cases, except for *O. sativa* and *O. brychyantha* in which intron 2 has been lost. The CDS and protein length of each analyzed SPO11 is shown in Table 2.

**Table 2:** **Length of the coding sequence and the respective deduced protein length of SPO11-1 and -2 from different species**.

**Organism**	**Gene**	**CDS length (bp)**	**Protein length (aa)**
*Arabidopsis thaliana*	SPO11-1	1089	362
	SPO11-2	1152	383
*Brassica rapa*	SPO11-1	1089	362
	SPO11-2	1143	380
*Carica papaya*	SPO11-1	1086	361
	SPO11-2	1149	382
*Oryza sativa*	SPO11-1	1146	381
	SPO11-2	1158	385
*Physcomitrella patens*	SPO11-1	1086	361
	SPO11-2	1113	370

Abbreviations: bp, basepair; aa, amino acid.

**Figure 2:**
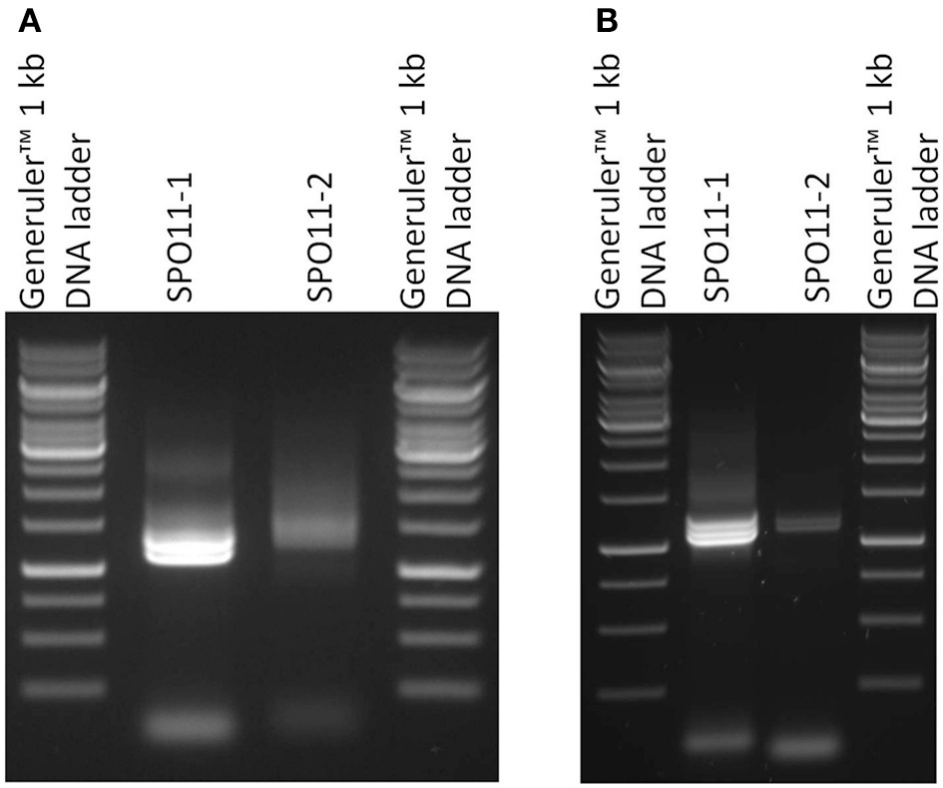
**Semiquantitative RT-PCR of SPO11-1 and -2 from *Arabidopsis thaliana* (A) and *Brassica rapa* (B)**. 1 μl of each cDNA was used for the PCR reaction. In the case of SPO11-1, distinct bands are visible. The lower band represents the α form of SPO11-1, the others are a mixture of other forms. The same holds true for SPO11-2.

Full length cDNAs were assembled from the RT-PCR data compared to the genomic sequences in the databases. Astonishingly, in our attempts to amplify the cDNA by RT-PCR for each gene we barely found one clearly distinguishable band. In most cases, more than one band accompanied with a smear was visible in the ethidium bromide stained gel (Figure 2). After cloning and sequencing of the PCR-products we were able to identify different alternatively spliced variants for both *SPO11* cDNAs.

### Pattern of alternative spliced SPO11

**Figure 3:**
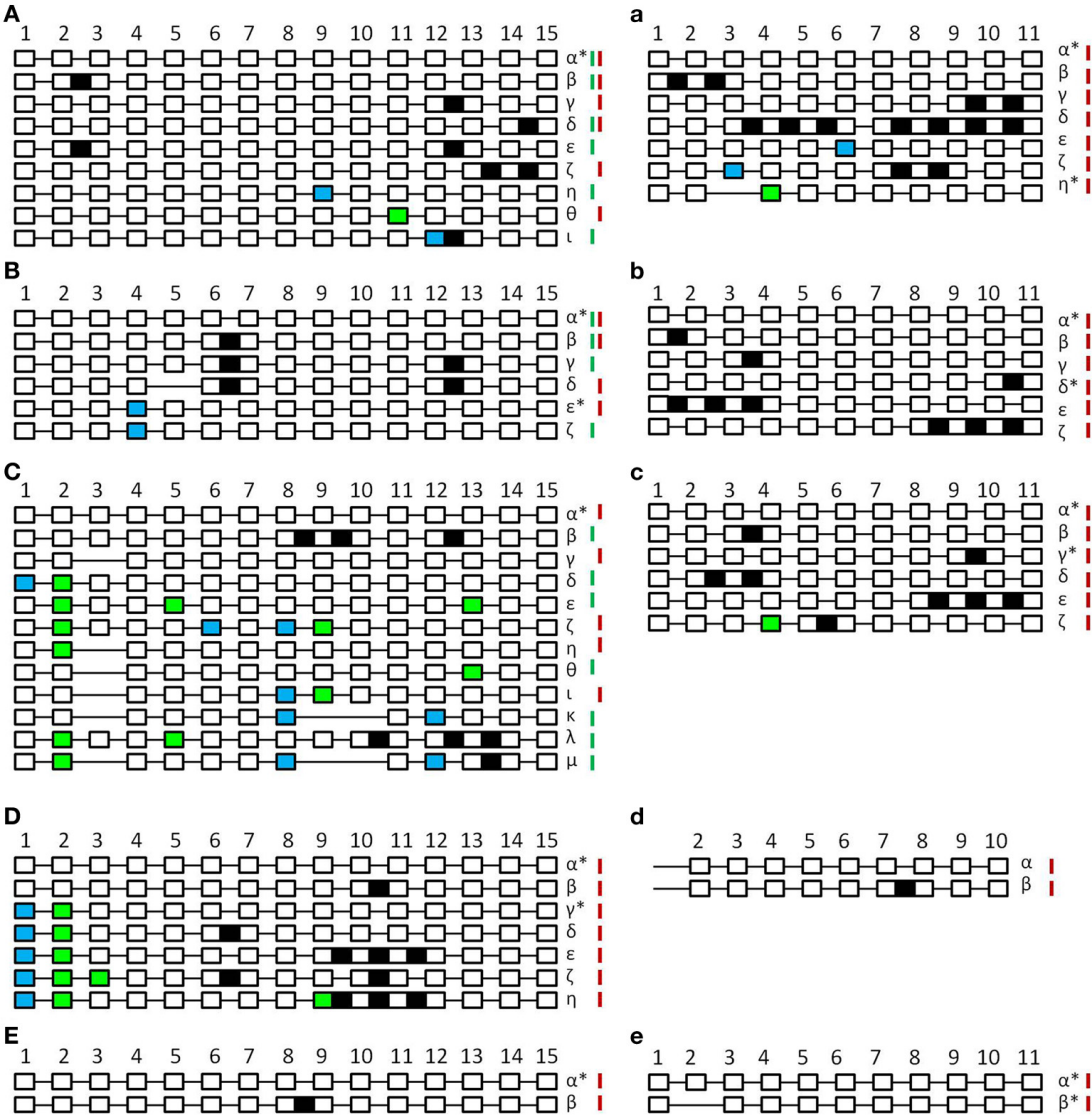
**Schematic unscaled schema of the different splice forms of SPO11-1 (A–E) and -2 (a–e) from *Arabidopsis thaliana* (A,a), *Brassica rapa* (B,b), *Carica papaya* (C,c), *Oryza sativa* (D,d), and *Physcomitrella patens* (E,e)**. Exons are numbered and shown as white blocks, spliced introns as black lines. Intron retention events are illustrated as black boxes, alternative 5′ splice site selection are shown as blue boxes and alterative 3′ splice site selection as light green boxes. In the case of exon skipping the corresponding white box is missing. Splicing forms are named in Greek letters. Splice forms found in generative tissue are marked with a red bar; splice forms found in vegetative tissue are marked with a green bar. Splice forms found in both tissues have both bars. Putative functional forms are marked with an asterisk. Due to high GC content and resulting PCR failure, amplification of OsaSPO11-2 was only possible from exon 2 so exon 1 is not indicated.

In the course of analyzing the patterns of alternative splicing events for *SPO11*, different splicing events which lead to putative non-functional proteins could be detected (Figure 3). In most cases we found intron retention (IR) mostly leading to a premature termination codon (PTC) and an altered length of the putative proteins. In some cases exon skipping (ES) occurred and we also observed events with altered 5′ or 3′ splice sites (alt 5′ss or alt 3′ss) leading to shorter or longer exons which led to the integration of PTCs in most cases.

When comparing the patterns of alternative splicing events of *SPO11-1* in vegetative and generative tissue we could only detect very few events with a matching pattern in both tissue types (Supplemental Table 4). Furthermore, these patterns are also different between the analyzed plants. We found no conserved alternatively splicing events between two different plants in our analyses, indicating that the events are species and tissue specific.

Analyzing *A. thaliana SPO11-1* (Figure 3A), a total of eight alternative splicing events could be found (β-ι). From these, five events were IR (β-ζ), one alt 5′ss (θ), one alt 3′ss (η), and one alt 3′ss combined with IR (ι). All alternative splicing events resulted in altered putative truncated proteins varying from 69 amino acids (aa) to 324 aa in length instead of 362 aa (Supplemental Table 4). For *A. thaliana SPO11-2* (Figure 3a), six alternative splicing events could be observed (β-η), three IR events (β-δ), one alt 5′ss (ε), one alt 5′ss combined with IR (ζ), and one alt 3′ss combined with ES (η). Five forms result in PTC and putative truncated proteins ranging from 52 to 305 aa instead of 383 aa. One form missing exon 3 and parts of exon 4 does not contain a PTC and is leading to a putative functional protein of 303 aa (η) (Supplemental Table 4).

The analysis of *SPO11-1* alternative splicing events in *B. rapa* revealed five different forms (β-ζ), which consist of two IR (β,γ), two alt 3′ss (ε,ζ), and one combination of ES with IR (δ) (Figure 3B). Leading to one alternative splicing event without PTC where the protein length is shortened by 9 aa (ε). All other events lead to PTC and therefore the putative protein sequences were truncated ranging from 82 to 153 aa instead of 362 aa (Supplemental Table 4). In the case of *B. rapa SPO11-2, five* alternative splicing events were detected (β-ζ). All of them had one or more IR (Figure 3b), four of them with a PTC putatively leading to truncated proteins between 32 and 268 aa length. One IR event, the retention of intron 10 (δ), did not lead to a PTC resulting in an altered putative protein with 404 aa instead of 380 aa (Supplemental Table 4).

The evaluation of the alternative splicing events in *SPO11-1* from *C. papaya* revealed the highest number of 11 alternative splicing events (β-μ), all differing in type (Figure 3C). We found IR, ES, alt 5′ and 3′ss as well as all kinds of combinations between those types. All constructs contained a PTC leading to putative truncated proteins ranging from 30 to 210 aa in size, instead of 361 aa (Supplemental Table 4). When looking at *CpaSPO11-2*, five different alternative splicing events were detected (β-ζ). All had IR but also one combination of IR with an alt 3′ss was detected (ζ) (Figure 3c). Four events lead to PTC and putative proteins between 97 and 270 aa instead of 382 aa. One event could lead to an altered protein with 410 aa in length containing intron 9 (γ) (Supplemental Table 4).

In *O. sativa* we were only able to analyze the alternative splicing events for *SPO11-1*, due to the fact that *SPO11-2* has a very high GC content in the 5′ region of its genomic coding sequence. This high GC content prevented successful amplification of the cDNA up to exon 2. In the case of *SPO11-1* we identified six alternative splicing events (β-η). We found IR as well as a combination of alt 5′ and 3′ss with and without IR (Figure 3D). Five of these constructs lead to PTC resulting in altered putative protein lengths between 109 and 237 aa instead of 381 aa. One construct with a shortened exon 1 and 2 did not lead to a PTC (γ) and results in a truncated putative protein with the length of 350 aa (Supplemental Table 4). Despite the problems with PCR amplification, we identified one alternative splicing event (Figure 3d), containing intron 7 for *SPO11-2*.

Looking at *P. patens*, we could only find one alternative splicing event for each *SPO11* (Figures 3E,e). In *SPO11-1*, intron 8 was retained resulting in a PTC and a putative shortened protein of 181 aa instead of 361 aa (Supplemental Table 4). In *SPO11-2*, exon 2 was skipped without causing a PTC, but generating a putative truncated protein with a length of 342 aa instead of 372 aa (Supplemental Table 4).

The majority of alternative transcripts found in these experiments lead to putative non-functional proteins. Only a small number of alternative transcripts may lead to functional protein forms these transcripts were exclusively found in generative tissue and were outnumbered by the alternative transcripts which contained a PTC.

## Discussion

### Evolution of different SPO11 genes

**Figure 4:**
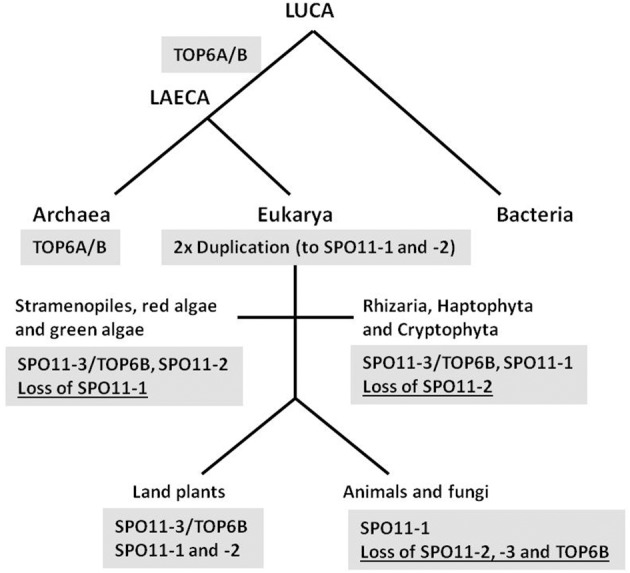
**Proposed evolution scheme of SPO11 by two duplications and different loss events**. The proposed evolution of the three different SPO11 genes nowadays found in land plants is shown schematically. Whereas bacteria do not possess a topoisomerase 6, LAECA has developed a topoisomerase type 6 from which the subunit TOP6A is orthologous to SPO11-3 in eukaryotes. Two duplication events of SPO11-3 took place after separation of eukarya from archaea resulting in the additional SPO11-1 and SPO11-2 genes. In different phyla loss events of either SPO11-1 or -2 occurred. After separation of the animal and fungal kingdom SPO11-2 and -3 as well as TOP6B must have been lost resulting in the remaining single SPO11 gene present in these two kingdoms. Abbreviations: LUCA, last universal common ancestor; LAECA, last archaeal-eukaryal common ancestor. The term LAECA was taken from Forterre (2013).

The time frame of *SPO11* gene evolution remains unclear as a second *SPO11* copy must have arisen very early, most probably by gene duplication and subsequent divergence of the two genes. The most likely scenario is that SPO11-3, which shows by far the best sequence homology to TOPVIA from archaea and additionally is still functional and interacting with TOPVIB in plants, was the ancestor of gene duplications giving rise to other *SPO11* copies (Hartung et al., 2002a; Malik et al., 2007). The phylogenetic sequence homology of SPO11-2 to the second SPO11 found in protists shown by Malik et al. (2007) favors this gene as the first result of duplication and speciation. However, as we could show earlier and sustain here, *SPO11-1* from plants is clearly orthologous to *SPO11* from fungi and animals, indicating a very early appearance of this gene by duplication of *SPO11-3* (Hartung et al., 2002a; Forterre et al., 2007; this work). Therefore, in our opinion a duplication of the ancestral *SPO11-3* must have occurred twice and very early giving birth to *SPO11-1* and *-2* that currently we can find either in animals and fungi (*SPO11-1*) or algae and protists (*SPO11-2*). The organisms that currently only contain *SPO11-1* must have lost the other copies, whereas protists that contain *SPO11-2* and *-3* orthologs have lost only *SPO11-1* (Figure 4). Finally, in land plants all known copies of *SPO11* are still encoded and active as we and others have show for all three *SPO11* genes earlier (Grelon et al., 2001; Hartung et al., 2002a,b, 2007; Sugimoto-Shirasu et al., 2002; Stacey et al., 2006). In addition, *SPO11-3* is present together with the second subunit *TOPVIB*, not only in plants but also in all so far investigated green algae and protists, which is not the case in animals and fungi (Malik et al., 2007) (Figure 4). This points to a conserved and linked function of both gene products together as we and others have shown for Arabidopsis (Hartung et al., 2002b; Sugimoto-Shirasu et al., 2002).

Nevertheless, the exact evolution and function of two SPO11 in plant meiosis is still enigmatic. We show that both meiotically active *SPO11* genes are undergoing an extremely complicated splicing procedure leading to high numbers of mostly aberrant alternative splice products. Despite the very high conservation of the gene structure for *SPO11-1* and *-2*, whose introns are in virtually 100% identical positions throughout all land plants, the alternative splicing seems to be regulated specifically in each species. It is not clear whether all different splicing forms of *SPO11* found in this study are real alternative spliced transcripts or if some may result from sampling unprocessed pre-mRNAs or genomic DNA contamination. However, there are some clues that the identified alternative splicing patterns are real events. (1) The pattern is found for both *SPO11* in a similar rate and the same as described by Hartung and Puchta (2000), (2) the pattern is conserved between different species, (3) amplification of genomic DNA was not possible (Supplemental Figure 3A) and (4) of the analyzed meiotic genes, only *SPO11-1* and *-2*, *PHS1* and *VIP3* show this pattern (Supplemental Figure 3B). An alternative splicing pattern was described for *VIP3* and *SPO11-1* earlier (Hartung and Puchta, 2000; Terzi and Simpson, 2009). This study is slightly differing in the findings for *SPO11-2* from the study done by Hartung and Puchta (2000), due to the fact, that we now took a closer look especially on *SPO11-2* and used a different protocol for RT-PCR combined with a higher number of PCR cycles. The conservation of alternative splicing between orthologous genes has been described in *A. thaliana* and *O. sativa* (Wang and Brendel, 2006). For this reason, it is not extraordinary that the alternative splicing is conserved not only between *A. thaliana* and *O. sativa* but also between the other analyzed species. Wang and Brendel (2006) also reported that the type of alternative splicing is more conserved than the respective intron which is spliced, also seen for *SPO11-1* and *-2* in this study.

Having a look at another kingdom in the eukaryotes previous studies showed also for mouse and human a pattern of alternative spliced transcripts for SPO11 (Shannon et al., 1999). In this previous work various alternative spliced transcripts were identified. Most of them were not further analyzed, but two transcripts variants with the expected size code for functional proteins. These two forms, SPO11-α and SPO11-β differ only in the abundance of exon 2. SPO11-α is missing exon 2 resulting in a shortened protein. The same forms were found in humans (Romanienko and Camerini-Otero, 1999). We were not able to find splicing forms equivalent to SPO11-alpha/beta from mammals due to the fact that the protein sequence in this area has not much homology to SPO11 from plants. But we were able to find other putative functional forms in plants as shown in Figure 3. The fact, that alternative splicing of *SPO11* is also common in other kingdoms, let us suggest that this mechanism is highly conserved and might have a regulating function.

### SPO11 and the NMD pathway

Many aspects are known to initiate non-sense mediated decay in plants. It was shown that long 3′ untranslated regions (UTRs) as well as an intron in the 3′UTR can trigger the NMD pathway (Kertész et al., 2006). We could previously show that *A. thaliana SPO11-1* and *-2* both harbor an intron in the 3′UTR and show different poly A sites, which sometimes results in long 3′UTRs (Hartung and Puchta, 2000). In this study we determined various poly A sites of *SPO11* in *O. sativa* and *C. papaya* (data not shown) that affect the position of the poly A tail and sometimes lead to long 3′UTRs. Another aspect which may lead to non-sense mediated decay besides a long 3′UTR are upstream open reading frames (uORFs) adjacent to the start codon of the gene (Nyikó et al., 2009). Analyzing the 5′UTR of *A. thaliana SPO11-1* and *-2*, we could identify in both cases long uORFs. For other species such as *C. papaya* and *O. sativa*, such long and adjacent uORF could not be found for both *SPO11*. However, for all analyzed species we were able to identify alternative splicing events that lead to PTCs which are presumed to be targeted by the non-sense mediated decay pathway (for recent review see Reddy, 2007). In plants, many pathways such as the circadian clock and the flowering time are controlled via alternative splicing of core genes (James et al., 2012; Staiger and Brown, 2013). Alternative splicing and various polyadenylation has been reported for *VIP 3* during flower development of Arabidopsis (Terzi and Simpson, 2009). VIP 3 is the Arabidopsis ortholog of SKI 8 from yeast, one of the described direct interaction partners of SPO11 in *Saccharomyces cerevisiae* (Arora et al., 2004). There must be a reason for the conserved alternative splicing of *SPO11-1* and *-2* in plants. One possibility could be that *SPO11* is controlled in a precise way via the pathways of alternative splicing and non-sense mediated decay. The NMD pathway offer a mechanism which is routinely used by mammals and others to regulate gene expression (Lareau et al., 2004; Lejeune and Maquat, 2005). Such effects were observed for mice and men where the splicing of *SPO11* and other meiosis specific genes are regulated during meiosis (Habu et al., 1996; Schmid et al., 2013). It has long been known for yeast that genes which are involved in meiosis show alternative splicing (Engebrecht et al., 1991; Guisbert et al., 2012). Considering that the number of possible NMD candidates in plants are quite similar to the frequency observed for humans, it seems likely that plants may also use non-sense mediated decay and alternative splicing for gene regulation in a comparable way (Lareau et al., 2004; Wang and Brendel, 2006).

While further analyses on the localization of the alternative spliced isoforms need to be done, this study revealed differences in the alternative spliced forms of *SPO11-1* and *-2* between generative and vegetative tissue. Such tissue specific regulation of NMD was shown before. Especially in mammals this has been studied recently (Zetoune et al., 2008; Huang and Wilkinson, 2012) An accurate differentiation between single cell types could give closer insight into the alternative splicing during pre-meiotic and meiotic stages as done for yeast and mammals (Engebrecht et al., 1991; Schmid et al., 2013). The very weak expression especially for *SPO11-2* could make this a challenging task. Up to now little is known about the function of the conserved domains in *SPO11* (Bergerat et al., 1997). A closer look and more information on those domains could contribute to the understanding of the putative function of the alternative spliced isoforms. Investigating *nmd*^−/−^ mutants could provide us with more information about the potential regulation of *SPO11-1* and *-2* via NMD in Arabidopsis. In previously published studies, *SPO11* mRNA was not captured mostly due to its weak expression and inadequate conditions for the amplification of *SPO11* (Simpson et al., 2008; Kalyna et al., 2012). Taking a closer look at *SPO11* expression in these plants would be of great advantage.

### Conflict of interest statement

The authors declare that the research was conducted in the absence of any commercial or financial relationships that could be construed as a potential conflict of interest.
