# Supplementary material for: The splicing fate of plant SPO11 genes
Source: Front Plant Sci. 2014 May 21;5:214. doi: 10.3389/fpls.2014.00214 (PMC4071758; doi:10.3389/fpls.2014.00214)
Supplement: Supplementary file 1 [file DataSheet1.ZIP › Supplemental Figure 2.DOCX]

Supplemental figure 2: Position of introns in SPO11 genes in relation to the deduced protein sequence in different organisms.

HsaSPO11_396 MAFAPMG--------------------------------------PEASFFDVLDRHRES---------------------------------------- 22 12 introns

CciSPO11_401 ------------------------------------------MDVFKDDVFWDINTARDP---------------------------------------- 18 13 introns

AthSPO11-1_362 ---------------------------------------------------------------------------------------------------- 0 14 introns

MguSPO11-1_361 ---------------------------------------------------------------------------------------------------- 0 14 introns

AthSPO11-2_383 --------------------------------------------------------MEESSG-------------------------------------- 6 10 introns

MguSPO11-2_373 --------------------------------------------------------MGELS--------------------------------------- 5 10 introns

AthSPO11-3_427 MADKKKR--KR--SKDD--------------E--AEELPFKSILESDDVITELLKSYISSSIKAAAGAGGASSS-------------SSKPL-------- 59 1 intron

GthSPO11_175505_410 MESSNTLLS-----------------------------LLLNRMKDFDDTYASIEEVFNA---------------------------------------- 31 7 introns

GthSPO11-3_76523_427 MLFVLTLIGRVGFPAFFG----------LVMSKKRRAEAADMSQEDMEETLKEVRTLARH-------LQQSS----------------QAAS-------- 59 10 introns

AkeSPO11_411 MNKNKSKQGNKRK---------------------SSTPPFQPGDLKHVKTLTKKEHLANG-----------------------------KLR-------- 42 2 introns

AanSPO11_52497_407 MAPKKKQKKAAAPEAEIL---------------------------DPEVVLKEVRRCRAL-------LATQK---------------------------- 38 0 introns

EhuSPO11-B_363 ---------------------------------------------------------------------------------------------------- 0 4 introns

EhuSPO11-3_436 MPPRKKAGASSQFDASEVMQPVQALRGRMDAA--GSSRKRASSQFDASEVMQPVQALRGR-------MDAAG---------------SSRKR-------- 68 13 introns

FcySPO11-3_239125_443 MSTKKRSRTVPPPVVDAAEASSLFIAASATGKKKNRKRVEVKLAADADIVLAKCRELKEK-------LRRKK-------REAAT---TGTPS-------- 75 2 introns

FcySpo11_242364_353 ---------------------------------------------------------------------------------------------------- 0 2 introns

GlaSPO11_368 ---------------------------------------------------------------------------------------------------- 0 0 introns

PhtSpo11-3_24838_430 MASRNRREPLASGPAPRPDMS-------ATST---RKRKAVALAADADLVLRKCRQLRQS-------LEAKA-------------EIESSVV-------- 62 2 introns

PhtSPO11-B_36531_432 ---------------------------------MEDLIESGVVEDGTNQGRYAVIDATLEIP-------------------------------------- 29 0 introns

PciSPO11_382 ---------------------------------------------------MATDRRPGAAT-------------------------------------- 11 14 introns

PniSPO11-B_391 --------------------------------MIEDLVLQTVIEPLSREPDPAVPEINASVD-------------------------------------- 30 2 introns

PniSPO11-3_446 MSRKKRSRTAADSTPTTPAAS------VAVGK--NRKREQVKLASDADLVLAKCRALKQT-------LLQKKDDPDSLARVGATGSIEGSGD-------- 77 1 Intron

TpsSPO11_263510_413 ---------------------------------------MLVVEENEEQ----LLRSTVQY--------------------------------------- 18 3 introns

TpsSPO11-3_425 MPPTKKPRVSGGAAVARTSKP--------STT--------VKLADDADEVLAKCRSLRDA-------LRMKA-------------S--STGD-------- 54 7 Introns

BnaSPO11_133434_398 -----------------------------------------MEVPTKNTAVSKIPAPSTP---------------------------------------- 19 8 introns

MbrSPO11-3_375 ---------------------------------------------------------------------MAN---------------QEAMK-------- 8 9 introns

CreSPO11-B_14g619800_263 ---------------------------------------------------------------------------------------------------- 0 9 introns

CreSPO11-3_413 MSKRGG--------ADD--------------K--ALALAKGKRIRGAEDVLKKVQALATQ-------LKNSK----------------EVEK-------- 45 14 Introns

CvaSPO11-3_418 MSKVVGK--KRPLTTDE--------------K--AAEAAK-RQIKSAEEVLQHVKKLTKL-------IEREP----------------RIAK-------- 50 9 Introns

CvaSPO11-B_423 ----------------------------MANELLAHIFGHEGAAARLAGESQWEEDGAALEE-------------------------------------- 34 6 Introns

HsaSPO11_396 -------LLAALRRGGREPPTGGSRLASSSEVLASIENIIQDIITSLARNEAPAFTIDNRSSWENIKFEDSVGLQMVSH--------CTTRKIKSDSPKS 107

CciSPO11_401 --------VSVLYAVEESERTDPMGDSGPYDDFEELGDGEEEKWPTSKQGSESEVDLE----EEQIDVVKCIEDMVLS------------FLKQIEACG- 93

AthSPO11-1_362 -------------------MEGKFAISESTNLLQRIKDFTQSVVVDLAEGRSPKISINQFRNYCMNPEADCLCSSDKPK-------GQEIFTLKKEPQT- 73

MguSPO11-1_361 -------------------MEGKHSIPNRLYLLTKIKELTRSLVEDLCNGRSPTLKIDQFTSYCSEDSGKCCCSYNLPK-------GKEILTLRRESQS- 73

AthSPO11-2_383 ---------LSSMKFFSDQHLSYADILLPHEARARIEVSVLNLLRILNS-------PDPAISDLSLINRKRSNSCINKGILTDVSYIFLSTSFTKSSLTN 90

MguSPO11-2_373 ----------KNSILFSDQHLCHADILPPSEVRARIEVAILNFLKNLNS-------SNPEVTDLPLISRKMGNSRVRRGLLTDDSKIFLSHSFSTKSLMN 88

AthSPO11-3_427 -------TLADLSLSSS---CREVADLSLSSVQTEIETVIVQIARSILAGDGFSFSVPSR-AASNQLYVPELDRIVLK---------DKSTLRPFASVSS 139

GthSPO11_175505_410 --------LDAFESSDPL-REYSIKDSQRELVLDRVLSIVAHCFRALANDKFPTIKYRSGQMYMDDSPNECLDFEDPKD---GFKFSSGVLSLRGRSSKN 119

GthSPO11-3_76523_427 -------SVSELGYTN----LFEVSDQSPEEVRKNIELVACQAVASILDGRGLAFTFAQRGSATNVQFVPELDRNYLE---------YKAMKRQFADSSQ 139

AkeSPO11_411 -------TLEQLGIKDD---VTEVKVMNEEEVSIRIEQLIIQCVTSILQGKGFEYRVPSR-STANQLYIPELDRIALKS--------DKITVRSFVNTAH 123

AanSPO11_52497_407 -------PVDMSTVDLSG--MRVVDAMPADDVAYAIEALFVAAAESIMRGESFKFDVPSR-SATNQRYVRELDRIVLK---------HNASERSFANVGQ 119

EhuSPO11-B_363 -----------------------------MSAQLAISSLTASARTRLAAGDARAMDLSLSRVDAHAAQIMQLTG-------------RTAHRPSVRGVAH 58

EhuSPO11-3_436 -------TLEEVGVSGV---SKEVQHMGAEDVSAEIEAICMRAVASIMRGDGFSYMMPSR-VASNQMYVKELDRVVLK---------EKMLERVFSNASS 148

FcySPO11-3_239125_443 -------DDDSNAIPKD---IVEVDELSTTEVLEGIEGVALQITRQVLQKKGFSMEIPSR-ASSNQIYLKDRDRLVLG---------GKRVGRSFLNVRE 155

FcySpo11_242364_353 ----------------------------------MVASFCHDLLAPSS--------------SSSVMNNEDDN-------NEEDS--DDDDDVDIED--- 40

GlaSPO11_368 -----------------MCDIDKLGHQDDRAILELYRRILLQLYEQVESS-----------AAGLILPMSEQD-------------LQDKKGISFKRHP- 58

PhtSpo11-3_24838_430 -------GVLPEGIPSD---IVEVVELPATQVLEGMEAMALHIAQQVLERKGFSLEIPSR-AASNQIYVKEWDRIVLG---------GKRSSRSFLNVKE 142

PhtSPO11-B_36531_432 ---------GRALSVTQTPVYGRHDHLTSDEVIARIERLIETVVLALERGRMPIL-ETL---WIPLENGSGGE-------DAVLG--AQQGDILRKTFH- 106

PciSPO11_382 ---------AGWIALSRDERPCYRDALWSGQVLRRIEALAADFLECLSTGKVLEL-EALKRDSSAMIYDHEKQ-------RQVHGNDKRLVKLNRQG--- 91

PniSPO11-B_391 -----------AIDFKRKLSLHHLTQARSLTSILKVAAFCYELLVPCT--------------IVAERETTEDR-------NSVAH--SDAGNIDEEEEG- 95

PniSPO11-3_446 -------DEDPDKIPKD---IVEVDELSATEVLEGIEGVALQITRQVLAKKGFSMEIPSR-SASNQIYVKEWDRIVLG---------GKRTGRNFMNVRE 157

TpsSPO11_263510_413 -------------TYSERVSYEQHDN-SPLDVIERIEAVVGSLVTYLGDMRGPVL-REY---LHPIVSTREDESSVDEEHEEVYHPPKEQLELFFEYFSN 100

TpsSPO11-3_425 -------NDDDSGTTADGKAIVEVVEMNTTQVMEGIENVAVKIAKQVLAKQGFQLEIPSR-SASNQVYVPELDRIVLG---------DKTGTRSFLNVKE 137

BnaSPO11_133434_398 ---------REIDGDDGEEFDIRNQTQTSREVSDCDSNSLRDSLASNDERISESDSESYSRFGSDQSNESDNESDLFDEWTSLFGDWKTQVGRESKSIVS 110

MbrSPO11-3_375 -------TIAELGYNDV---T-EVLEMDDEEVAQRIETIATDAVRAIMGGQGINYVMPSR-ANNNQKYIPELDRIVLL---------NAVKTRAFDSLSS 87

CreSPO11-B_14g619800_263 ---------------------------------------------------------------------------------------------------- 0

CreSPO11-3_413 -------TLAELDLPYN---CREVVDKDGDDVMGEIEAAMYNVAASILGGEGFAFDVPSR-AKGNQMYVPELDRIVLR---------DAVSKRPFASTAT 125

CvaSPO11-3_418 -------SLAALDLPQA---CREVVDKGASAVLDEIEAAIYMVASSILRGEGFSYDIPSR-AKGNQLYVPELDRIVLK---------DSTSQRPFASTAT 130

CvaSPO11-B_423 ---------ADEPGQGSGGDPGLDMDASPEEVVRRIQSATLWFVSQLCQGQLPDIEPVQRDAGNRALAQSAAGGEDEEGEVGGGPHVLRMQQLTQRRSLV 125

HsaSPO11_396 --------------AQKFSLILKILSMIYKLVQSNTYATKRDIYYTDSQ----LFGNQTVVDNIINDISCMLKVS------RRSLHILSTSKGLIAGNLR 183

CciSPO11_401 ------------AVILDDAQLFRVLDIMHEGLVTGTPTTKRDIYYKDVA----LFGRQSVVDTLVDDLAATWGTQ------RCDLNVRAASKGLVCGAGI 171

AthSPO11-1_362 ---------------YRIDMLLRVLLIVQQLLQENRHASKRDIYYMHPS----AFKAQSIVDRAIGDICILFQCS------RYNLNVVSVGNGLVMGWLK 148

MguSPO11-1_361 ---------------RRFDALLRVLLIVQQLLQENKHGSKRDIYYMHPS----VFKEQSVVDRAVNDICILLQCS------RHNLNVVSVGNGLVMGWVR 148

AthSPO11-2_383 -----------AKTAKAFVRVWKVMEICFQILLQEKRVTQRELFYKLLCDSPDYFSSQIEVNRSVQDVVALLRCS------RYSLGIMASSRGLVAGRLF 173

MguSPO11-2_373 -----------ENTANSFIRVWKVMEMCYQILVQEKKVTQRELFYKLLCDSPHYFTSQLQVNRTIQDLVALVRCS------RYSLGIMASSRGAVAGRLL 171

AthSPO11-3_427 --------------VRKTTITTRILALIHQLCLRNIHVTKRDLFYTDVK----LFQDQTQSDAVLDDVSCMLGCT------RSSLNVIAAEKGVVVGRLI 215

GthSPO11_175505_410 --------------LLRCARFFRVAQLVCSLLKANKTSTKRDIFYADGK----VFGKQINSDKAIELLARICNVP------RFQLNIVSYSKGLVYGALV 195

GthSPO11-3_76523_427 --------------VKRTTMMTRLMQLVHELAKKNIHSTKRDLFYADVK----LFEKQQNSDAVLEELSLMFGCT------RHSLQVTASEKGLVVGRLQ 215

AkeSPO11_411 --------------VKKTAVTTRVMQLIHNVLNKNIHITKRDLFYTDVK----LFVDQAASDGVLDDVACMLGCT------RTSLNVVASEKGVVVGKLR 199

AanSPO11_52497_407 --------------VRKTAIMTRVFSLVHEVVTKGIHVTKRDLFYTDVK----LFKKQEESDAVLDDAACTIGCP------RSCLNVVAADKGLVVGRVQ 195

EhuSPO11-B_363 ------------EQLRRLAQLWLVLAALYRNLVSGKKLAQRELWYRLKPLG--IFTSPQQVYDRVSESGAIIAEWTGKACPRESLGVIAAPRGSMTGTVS 144

EhuSPO11-3_436 --------------ARKTAITTRLMQIVLELCSKGIHVTKRDLFYTDVK----LFKQQTESDDVLDDVACMVGCT------RSSLNVVASEKGIVVGKLI 224

FcySPO11-3_239125_443 --------------SRKSAITLRVMQLLHAVLVKRIHITKRDLFYTDVK----LFVDQAESDGVLDDVATMIGCT------RSNLHVVASDKGLVVGRIQ 231

FcySpo11_242364_353 --------------GNYYYHRRRQNGRRR-----RRTTTTREVYYFFVT----HFRDQRECDKAIWDLVSILNLPS-----RQSLGLVASPKGWFCGSID 112

GlaSPO11_368 ---------------LRFARALRMIAESACAVVKKTFVNIRSLYYTAPS----LYGSQIQSNVLIAEFCFGYSIE------REMMHYRAAAKGLLLGNCT 133

PhtSpo11-3_24838_430 --------------SRKSAITLRVMQLLHAVLMKRIHITKRDLFYTDVK----LFVDQAESDGVLDDVATMIGCT------RSNLHVVASDKGLVVGRIQ 218

PhtSPO11-B_36531_432 -----------LHQCRSFTSILLVLDFCHSLLRARRTTTTREVYYYHVT----HYRSQKECDSAIQDTAILLQVP------RSSLGLKASPKGWFCGDVQ 185

PciSPO11_382 --------------GRRYTGIWLILQTAHALLTENKTATQRDVYYLHP-----FFKGQSEADEAILDAGSILGVP------RGCMNIVGATKGCFTGDIS 166

PniSPO11-B_391 ------------ARGTNGGPHRRRNGHRKLLQIPQRTTTTREVYYFYVT----YFRDQRECDKAIWDLVCILGLPS-----RQSLGLVASPKGWFCGSID 174

PniSPO11-3_446 --------------SRKSAITLRVMQLIHAVLVKRIHITKRDLFYTDVK----LFVDQAESDGVLDDVATMIGCT------RSNLHVVASDKGLVVGRIQ 233

TpsSPO11_263510_413 ATGKDVKIFDDYRTDRTFTSITKVMAFIHQLLLSNRTTTTREVYYVFVT----HFRNQKECDGTILDVAKILNVP------RRALGLSASPKGWFCGCVE 190

TpsSPO11-3_425 --------------SRKSAITTRVLQLLHAVLLKRIHITKRDLFYTDVK----LFVDQSESDGVLDDVATMIGCT------RSNLHVVASDKGLVVGRIS 213

BnaSPO11_133434_398 KLN--------ERTLMKHGRILLCLKAIYRNMKTNSRVTQRDLYYQFIQN----FESQNQLNETIYHVSCILWLP------RHELGIVATPRGLVFGDVA 192

MbrSPO11-3_375 --------------VRKTAIMTRVFQLIHQVVRKRIHVTKRDLFYTDVK----LFTKQEESDAVLDDVACMVGCT------RTSLNVVASDKGVVVGCVT 163

CreSPO11-B_14g619800_263 ----------------------------------GRTATQRELYYTLLR--PPLFASVRDVNAAVSDVAALLAVP------RAGLGLSASGRGAVAGRLL 58

CreSPO11-3_413 --------------CRKAAVTTRILGLVHELLGKNIHVTKRDLFYTDVK----LFEDQSQSDAILDDLACMLGCT------RSSLNVVASEKGVVVGRLS 201

CvaSPO11-3_418 --------------CRKAVFTTRVLGLVHELCMKQIHVTKRDLFYTDVK----LFEDQGNSDAILDDVACFLGCT------RSSLHVVASEKGVVVGRLT 206

CvaSPO11-B_423 GRH--------PESAEAVARLWVLLEAVHEMLLAGLQATQRELWYRFKT--LEVFRSPRDVGEAIQDAVGMLQVP------RSALGITASSKGLVAGRLV 209

HsaSPO11_396 YIE---EDGTKVNCT-------------------CGATAVAVPS-----------NIQGI------RNLVTDAKFVLIVEKDATFQRLLDDNFCNK-LS- 242

CciSPO11_401 TIN-LYSGEVVQGN---------------------DTEPSNIPAG---------EDIESFG-------VDEDIAWVLVVEKEAVFQTLCQLGVTHGLNI- 232

AthSPO11-1_362 FRE---AGRKFDCLN-------------------SLNTAYPVPV-----------LVEEV------EDIVSLAEYILVVEKETVFQRLANDMFCKT--N- 206

MguSPO11-1_361 FAE---AGRKFDCIS-------------------SPNTAHQIPV-----------YVEDV------EDIISVAEYILIVEKESVFQRLANDQFCRK--N- 206

AthSPO11-2_383 LQE--PGKEAVDCS------------------ACGS-SGFAITG-----------DLNLLDNTIMRTD----ARYIIIVEKHAIFHRLVEDRVFNH--I- 234

MguSPO11-2_373 LQE--PNQEIVDCS------------------TCGS-SGYVISG-----------DLNLLENLVMKSD----ARYIIVVEKHAIFQRLAEDRVFNQ--I- 232

AthSPO11-3_427 FSD---NGDMIDCTK-------------------MGMGGKAIPP-----------NIDRV------GDMQSDAMFILLVEKDAAYMRLAEDRFYNR--F- 273

GthSPO11_175505_410 MKD---LQGNLIHCH-------------------SGPTTLPS------------------------ELFVDQAHALFVVEKECIFRRLCDG--------- 240

GthSPO11-3_76523_427 FND---DDDFIDCTK-------------------MGRSGKNIPS-----------FLDRV------SNIKSDSEFILLVEKDAAFQRLAEDRFYNT--Y- 273

AkeSPO11_411 FVD---DGDPIDCSR-------------------MGAGGKAIPP-----------FTDRL------TNIESEAKFILLVEKDAAFMRLAEDRFYNS--Y- 257

AanSPO11_52497_407 FDE---DGDPIDCTR-------------------MGVTGKAIPA-----------YIDKI------SNIRSDAEFILLVEKDAAYNRLAEDRFYNQ--F- 253

EhuSPO11-B_363 LATP-DGPRQLDD------------------------AVYAIPG-----------DPEECS---RLRFASSRARYVVVVEKDTVFTRLLDDRFTR--LL- 202

EhuSPO11-3_436 FED---DGDTIDCTK-------------------MGMGGKAIPP-----------SIDRV------TNVRGDAQFILLIEKDAAFMRLAEDRFYND--Y- 282

FcySPO11-3_239125_443 FEE---DGDFIDCTK-------------------MGVGGKAIPP-----------YIDKI------ENIRSDAEFILLVEKEAAYMRMAEDRFYNK--Y- 289

FcySpo11_242364_353 VYNGHTNELKFNGR------------------ELD-THGMAITPSTDPMDDDDNNGNGNGNDNIRIESD--AR-CILVIEKEGVYTRLSEDKFFLNY-L- 188

GlaSPO11_368 MVT---QVGTVTAS---------------------SLVAVPISP-----------HFFYL------QQIHTDAQFVLVIEKDTVFEHLIDSYEAIKDRLG 192

PhtSpo11-3_24838_430 FEE---DGDFIDCTK-------------------MGVGGKAIPP-----------YIDKI------ENIASDAEFILLVEKEAAYMRMAEDRFYHR--Y- 276

PhtSPO11-B_36531_432 LVS--NGQVVLDGR------------------HLQSIHGAPISG----------EWLAPTR-DFTIHSC--AATCILVIEKEGVYNRLVEDRFFDR--F- 249

PciSPO11_382 ILVR--GSTPRDGS------------------WRHFGSGEEVS-------------ITQELLQLKPCDIN-AK-FILVVEKDGIFNRLREDKFYEV--V- 228

PniSPO11-B_391 VYNAHTGELKFNGR------------------ELD-AHGTAITP---------NNNISSN--HIRIESD--AK-CILVIEKEGVYTRLSEDKFFLRY-F- 239

PniSPO11-3_446 FEE---DGDFIDCTK-------------------MGVGGKAIPP-----------YIDKI------ENITSDAEFILLVEKEAAYMRMAEDRFYHK--Y- 291

TpsSPO11_263510_413 ITR--RGTLSSGKD------------------VSGSIDGTALSSI---------QGLPITR-EWTEH----AK-VIVVIEKEGVYNKLSEERIFDD--F- 252

TpsSPO11-3_425 FFE---DGDFIDCTK-------------------MGVGGKAIPP-----------YIDKI------ENIQSDAEFILLVEKEAAYMRMAEDRFYQR--Y- 271

BnaSPO11_133434_398 MEC---DEERWDCR----------------------STRQLITS----------SVMNSN-----FTCSSFGAKAIIVVEKEGIFNRLVEDNFIKR--V- 249

MbrSPO11-3_375 FLE---DGDFIDCTR-------------------MGVGGKAIPS-----------LVNKI------TDIKGTAQFVLLVEKEAAFMRLAEDRFYQR--Y- 221

CreSPO11-B_14g619800_263 LREG-PAAPWLDCS------------------TAP--AGRPLPG-----------DLTAIGRGGGSGSGSGSGVYLVVVEKDAVFQRLAEDRLWEQ--L- 123

CreSPO11-3_413 FRE---DGDFIDCQR-------------------MGVGGKAIPP-----------NIDKV------SDISSDASFILLVEKDAAFMRLAEDRFYNT--Y- 259

CvaSPO11-3_418 YRE---DGDLIDCQR-------------------MGVGGKAIPP-----------NVDKV------TDIQSDALFILLVEKDAAFMRLAEDRFYNT--Y- 264

CvaSPO11-B_423 VHDQ-RAGTETDCA------------------ALGG-AGMQIPG-----------DIAHISRHYAYQS---DAQLVVVVEKDAVFQRLVQQRFFDA--V- 272

HsaSPO11_396 -PCIMITGKGVPDLNTRLLVKKLWDTFH-----VPVFTLVDADPHGIEIMCIYKYGS-------MSMSFEAHHLTVP--AIRWLGLLPSDLKRLN----- 322

CciSPO11_401 GKGIMVTGKGYPDIATRHLVKSLGDALPKH---VALAALVDCDPYGIDILSVYRYGS-------QSMRHENDTLATR--RIKWLGLRISEVMEWD----- 315

AthSPO11-1_362 -RCIVITGRGYPDVSTRRFLRLLMEKLH-----LPVHCLVDCDPYGFEILATYRFGS-------MQMAYDIESLRAP--DMKWLGAFPSDSEVYS----- 286

MguSPO11-1_361 -RCIVITGRGYPDVPTRRFLHLLIEKLR-----LPVYCLVDCDPYGIDILATFRFGS-------MQMAYDAKSLRLP--EIQWLGAFPSDCEKYQ----- 286

AthSPO11-2_383 -PCVFITAKGYPDIATRFFLHRMSTTFPD----LPILVLVDWNPAGLAILCTFKFGS-------IGMGLEAYRYACN---VKWIGLRGDDLN--L----- 312

MguSPO11-2_373 -PCILITAKGYPDIGTRLLLHRMSREFPE----LPILGLVDW---------IFKFGS-------IAMGLEAYRYACN---VKWLGLRKDDIEQ-L----- 302

AthSPO11-3_427 -PCIIVTAKGQPDVATRLFLRKMKMELK-----LPVLALVDSDPYGLKILSVYGCGS-------KNMSYDSANLTTP--DIKWLGIRPSDLDKYK----- 353

GthSPO11_175505_410 -RAILLTGCGYPDVQTRILLSRLSKSHPN----LPIYGIADWDPDGVEIILTYAFGS-------HSREHEREFLRCP--TMRWLGLHSNDMKVFG----- 321

GthSPO11-3_76523_427 -PCVIITGKGEADLATRMFLRRVKDALK-----IPILGLFDSDAHGLKILSVYMQGS-------EAMSHDSANLATP--DIKWLGVRPSDLDKYN----- 353

AkeSPO11_411 -PCIIITAKGQPDVATRMFLKRLREELK-----IPVLGLVDSDPYGLKILSVYMSGS-------KNMSYDSASLTTK--GIKWLGVRPSDLDRYN----- 337

AanSPO11_52497_407 -PCILITGKGQPDVATRMFLSRLKAELK-----IPVLAFVDSDPYGLKILSVYMSGS-------KAMSYDANSLTTP--DIKWLGLRPSDLDAYN----- 333

EhuSPO11-B_363 -PCLLITACGYPSLAVRALVQHVVKALA-----LPCVVLTDYNPHGMALMLCYKHGS---------ATFAMDGYCCP--ELKWVGLHTADVQLLETPTAE 285

EhuSPO11-3_436 -PCVIISGKGQPDVATRLFLNKVRSALN-----VPILGLFDADPYGLKILSVYMKGS-------KNMSYDSINLTTP--DIKWLGVRPSDLDKYS----- 362

FcySPO11-3_239125_443 -PCIVITAKGQPDVASRMFLSRITNELQ-----IPVLGLVDSDPYGLKILSVYMSGS-------KNMSYDSASLTTP--NIMWLGLRPTDLDKYD----- 369

FcySpo11_242364_353 -PCILVTGKGFPDIATRRWVRRMQKTLK-----IPVYGLCDCNPYGVSVLDTYRYDKGVKICNVEKRRKKKSDDEDDPLEIQWIGLRPSQIEHMN----- 277

GlaSPO11_368 QSFMVITGKGYPDAATRALVSFLDSCR------IPIFGLADGDAHGMNILCTYAYGSAS----ATRKSVVPNHYIAP--SLVPIGVFNSHIQRSS----- 275

PhtSpo11-3_24838_430 -PCIIITAKGQPDVATRMFLARITSELK-----IPVLGLVDSDPYGLKILSVYMSGS-------KNMSYDSASLTTP--DIKWLGLRPSDLDKYD----- 356

PhtSPO11-B_36531_432 -PCILVTGKGFPDLSTRALVHVLHHTLGL----LPVRGLCDCNPYGVMVLHTYQHT--------ARKGVDGGHRFGVP--ISWIGLRPSQVQQLQRQPNT 334

PciSPO11_382 -PSILITGRGFPDLATRVFVSLLSHCLE-----IPVLGLCDCNPFGLSIMLTYKLGS-------ARMPLESLEYAVD---IKWVGVRPSQVVGLG----- 307

PniSPO11-B_391 -PCILVTGKGFPDIATRRWVKKMQKTLN-----IPVYGLCDCNPFGVSVLDSYRHDQGN-----QHQSENRSDPLDD---LHWIGLRPSQVETMD----- 320

PniSPO11-3_446 -PCIVITAKGQPDVASRMFLSRITNQLK-----IPVLGLVDSDPYGLKILSVYMSGS-------KNMSYDSASLTTP--NIMWLGLRPSDLDKYD----- 371

TpsSPO11_263510_413 -PCILVTGKGFPDLATRALVNTLHKELD-----IPVVGICDSNPFGISVLALYYCAG-------ERMGVDGRMKYTVP--MMWIGLRPSTVESLEDD--- 334

TpsSPO11-3_425 -PCIVITAKGQPDVATRMFLSRITSELQ-----IPVLALVDSDPYGLKILSVYMSGS-------KNMSYDSASLTTP--DIKWLGLRPSDLNRYD----- 351

BnaSPO11_133434_398 -PSVIVTGLGYPPLYVRVLVHKIETKLR-----LPVFGLFDHGPHGMAIHLTYKAGS---------RRYGDLYAIKN---MKLVGLLSKDLSELK----- 326

MbrSPO11-3_375 -PCVIVTGKGQPDVGTRQFVRAVKDALN-----VPILGLFDSDPYGLKILSVYMSSS-------KNMSYDSASLTCP--DIKWLGLRPTDLDRYK----- 301

CreSPO11-B_14g619800_263 -PCVLVTAKGVPDIATRAFAARLAATFPG----MQPVGLVDFNPAGVVILATYKYGS-------DRMGPEGRAHPLPR--LRWLGVRGRHLA--G----- 202

CreSPO11-3_413 -PCIILTAKGQPDVATRLFLKKLKSSLK-----IPVLALVDSDPYGLKILSVYMKGS-------MNMSYDSSNLTTP--DIKWLGVRPSDLDRFD----- 339

CvaSPO11-3_418 -PCIILTAKGQPDVASRLFLRKLRDTLR-----IPVLALVDSDPYGLKILSVYMKGS-------MNMSYDSSNLTTP--DIKWLGVRPSDLDRFN----- 344

CvaSPO11-B_423 -PCILATGKGVPDLATRAFLSGLSEAFPD----LPLVGLVDWNPAGANILCVYRFGS-------ARMG-ESQHYALRT--LGWLGARSSQLQ--Q----- 350

HsaSPO11_396 ------VPKDSLIPLTKRDQMKLDSILRRP---------------YVT----CQPFWRKEMEIMADSKMKAEIQALTFLS------SDY---LSRVYLPNKLKFGGWI 396

CciSPO11_401 ------LDPSLLLPLTDYDENKIRSMLKRP---------------HLP-----R-RWRDELEATQARGRKAEIEVLCSLPRDQALRIGVGDSMNNPLLAYVEQKLHSLISRAS 401

AthSPO11-1_362 ------VPKQCLLPLTEEDKKRTEAMLLRC---------------YLKR---EMPQWRLELETMLKRGVKFEIEALSVHS------LSF---LSEVYIPSKIRREVSSP 362

MguSPO11-1_361 ------LPQHCLLPLTTEDKQKAEGILHRC---------------YVER---EAPSWRYELQLMLDRGVKFEIEALSVHS------LTF---LSENYLPSKIQMGAFI 361

AthSPO11-2_383 ------IPEESLVPLKPKDSQIAKSLLSSK---------------ILQEN------YIEELSLMVQTGKRAEIEALYCHGY----N--Y---LGK-YIATKIVQGKYI 383

MguSPO11-2_373 ------VPEESLIPLKPRDHQIAKSLSSSE---------------ILQDK------YKEELSFMVQSGQRAEIEALYFHGF----D--F---LAK-YIGKKIVQADYI 373

AthSPO11-3_427 ------IPEQCRLPMTEQDIKTGKDMLEED---------------FVK----KNPGWVEELNLMVKTKQKAEIQALSSFG------FQY---LSEVYLPLKLQQQDWL 427

GthSPO11_175505_410 ------VPKALRLELSSRDRARISRMCERK---------------VIK----ENPRWRNELEEMLIQDSKYEIECLNAISQGGSNSLSN---LSNHYIHDKICRFDYLTLNFATSVT 410

GthSPO11-3_76523_427 ------VPRECRLELTTHDVSFAESLKEKP---------------YIQ----KRSAWVKELETLLSRKEKAEIQAFTSKG------FQY---LTQEYLPRKLREGDWV 427

AkeSPO11_411 ------IPEQCRLAMTENDIKTGKAMLQED---------------WVQ----KNPEWVKELNLMIRTKKKAEIQALSSFG------FQY---LTQVYLPQKIKRGDWI 411

AanSPO11_52497_407 ------LPDQCRLPMTPKDIEMGKQLLKED---------------FVL----KNKQWVKELERMLHTKQKAEIQALSAFG------FQF---VTQVYLPRKLRQGDWI 407

EhuSPO11-B_363 TPGRSVLAATSFQPYSDRDGAIVDGLLRNP--------------VVAN-----NEQLRYEAEAMRADRRKLELEALHALG------PEF---FAY-FLRDKIISSCA 363

EhuSPO11-3_436 ------IPQQCRLEMSEHDLKTGRELLEED---------------FIK----ANPKWHRELELMVKTKVKAEIQALSSFG------FQY---LSKTYLPRKLKEGDWI 436

FcySPO11-3_239125_443 ------LPDQCRLDMTENDIKTGKELLNEE---------------FIQ----KNPKWMKEIENMIKTKKKAEIQALSSFG------FQY---ITEEYLPRKLREGDWI 443

FcySpo11_242364_353 ------LPSDVFQELTNNDKKRLESLLISSKSKSRTTPKNKNSKSFAERGGWNKEERVRELQAMYK--YKVELEALHWKG-----MD-Y---M 353

GlaSPO11_368 ------KAIKGSRHCDPADLKLYDILITR---------------LTALG----RLEWANEVQNLAALGMQYELETILGQE------GGLVSYVVETVVGVRKRICNFDNKCTATRLAGARANSS 368

PhtSpo11-3_24838_430 ------LPEQCRLDMTENDIKTGKEMMKED---------------FIQ----KNPEWMKELEIMVKTKKKAEIQALSSFG------FQY---VTEQYLPRKLKEGDWI 430

PhtSPO11-B_36531_432 KHGQSKLPDQVFQSLTALDKRRLEHHLLS---------EQHGWTTFGP-----DERRVEELEEMLKNGYKMELEALNWLG-----MD-F---ITK-WLGDIFHYQDRAGHGHEGNSCWMDII 432

PciSPO11_382 ------LPQSSFKALTRKDIAVAESLLQAN---------------FVQANKHYRGEVQMWLGDSLP--WKMELEALHFLG-----FT-F---LTS-FLQDCIQEGSFL 382

PniSPO11-B_391 ------LPSQVFQELTTNDKKRLQSLLV---------PRSTHSGSFAERGGRDKAERIRELKAMRN--YKVELEALHWKG-----SD-Y---LCQ-FV 391

PniSPO11-3_446 ------LPDQCRLDMTENDIKTGKELMTED---------------FIQ----KNPKWMKELETMVKTKKKAEIQALSSFG------FQY---ITEEYLPRKLREGDWI 445

TpsSPO11_263510_413 ------LPKDVFQSLTDLDYKRIDS-LLD---------EDN----LFL-----NEERYEEIVKMKESGKKVELEALYWLG-----SD-Y---MSN-WVVGMLREMD---DGNE-----MVAI 413

TpsSPO11-3_425 ------LPDQCRLNMTENDIKTGKELLQEA---------------FIM----KNPKWMKELQIMVKTKQKAEIQALSSFG------FQY---ITEVYLPRKLKEGDWI 425

BnaSPO11_133434_398 ---------LSTHPLTKRDVQLANSLLAEHP--------------LVR----ENDNYSSELREMLKSNRKAELQSLYENS------CRF---LADEFLPSRILQRRFI 398

MbrSPO11-3_375 ------IPEQCRLPMTAEDIETGRRLLQAD---------------FIT----KNEAWVKELRLMLDTKVKAEIQALSNFG------FQY---LTEVYLPQKLERGDWI 375

CreSPO11-B_14g619800_263 ------VGEAHLQRLTARDTALIRRSLRER---------------LSAAEP----GWVAELDAMEQAGYKGDIEALYHAAG----G----------------DTGG 263

CreSPO11-3_413 ------IPQQCRLPMTEEDIKTGKRLLEED---------------WIK----SNPEWVRELELMLASKVKAEIQALSSFG------FQY---LSQVYLPLKLQEGDWI 413

CvaSPO11-3_418 ------IPQQCRLPMTDEDIRTGKRLLEED---------------FIQ----ANAEWVKELEIMIASKVKAEIQALSSFG------FQY---LSQVYLPLKLQEGDWI 418

CvaSPO11-B_423 ------ADAGAFQELTARDRSMAAN-LSAV---------------LRGAAP----EWAAELGRMLSSGSKAEIEALETGEG----GA-D---LAD-LLVGYLERGDCI 423

**Supplemental figure 2:** **Intron position alignment.** The sequences of 29 different SPO11 proteins has been aligned using MegAlign (ClustalW accurate/slow gonnet). In the resulting alignment the position of all identified introns (if any present) of SPO11 genes has been manually incorporated using a colour code to indicate also the phase of the respective intron. Colour code: yellow = the intron is located between two coding triplets (phase 0) behind the coloured amino acid; green = the intron is located right after the first nucleotide of the coding triplet (phase 1) of the coloured amino acid; the intron is located right after the second nucleotide of the coding triplet (phase 2) of the coloured amino acid respectively. The length of the respective proteins in amino acids is given once in the name and at the end of the alignment lines. At the end of each line the position of the last amino acid shown in the respective line is displayed. At the end of line one the total number of introns for each organism is given.

Abbreviations: Aan = *Aureococcus anophagefferens;* Ake = *Aplanochytirum kerguelense;* Ath = *Arabidopsis thaliana;* Bna = *Bigelowiella natans;* Cci = *Coprinopsis cinerea;* Cre =*Chlamydomonas reinhardtii;* Cva = *Chlorella variabilis;* Ehu = *Emiliania huxleyi;* Fcy = *Fragilariopsis cylindrus;* Gla = *Giardia lamblia;* Gth = *Guillardia theta ;* Pci = *Phytopthora cinnamomi;* Pht = *Phaeodactylum tricornutum;* Pni = *Pseudo-nitzschia multiseries CLN-47;* Hsa = *Homo sapiens;* Mbr = *Monosiga brevicollis;* Mgu = *Mimulus guttatus;* Tps = *Thalassiosiria pseudonana*
