# Supplementary figures and images for: The splicing fate of plant SPO11 genes
Source: Front Plant Sci. 2014 May 21;5:214. doi: 10.3389/fpls.2014.00214 (PMC4071758; doi:10.3389/fpls.2014.00214)

A

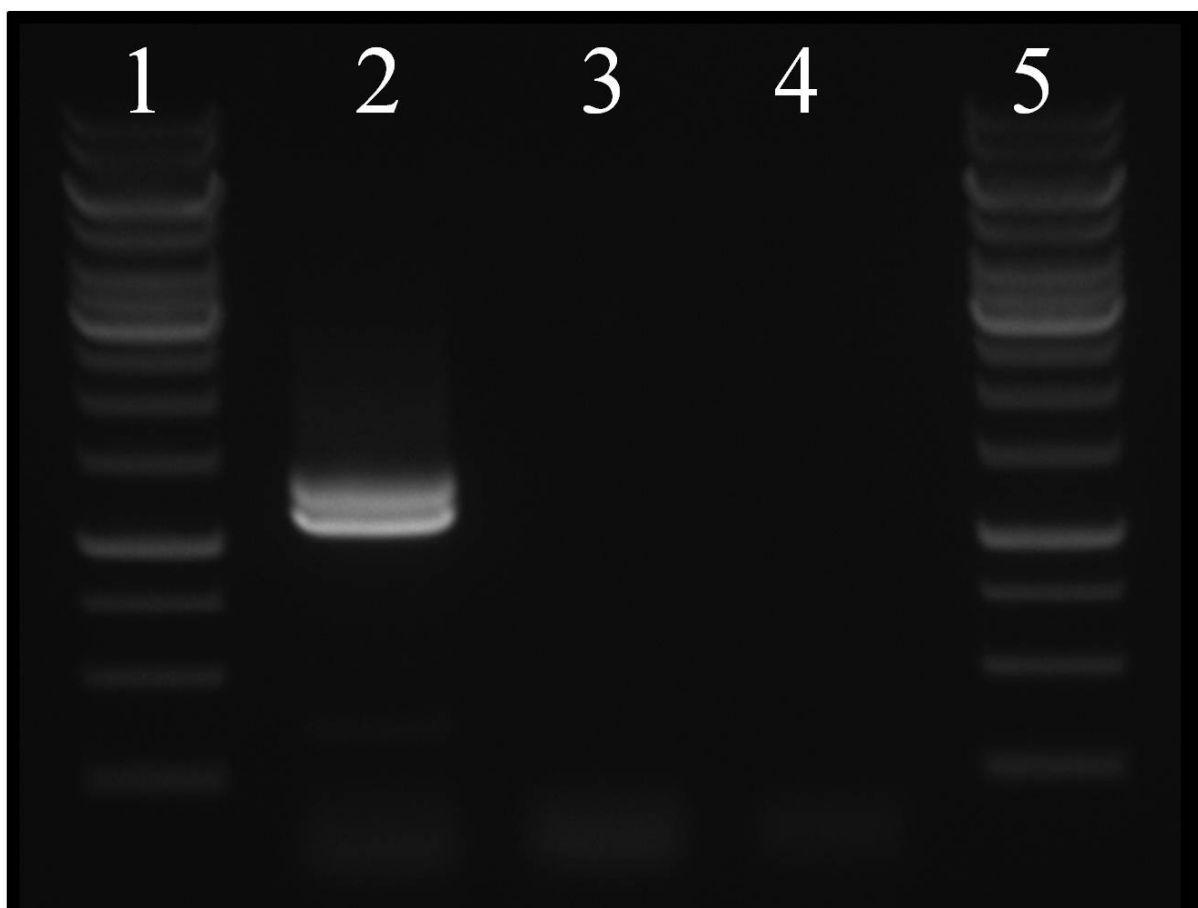

B

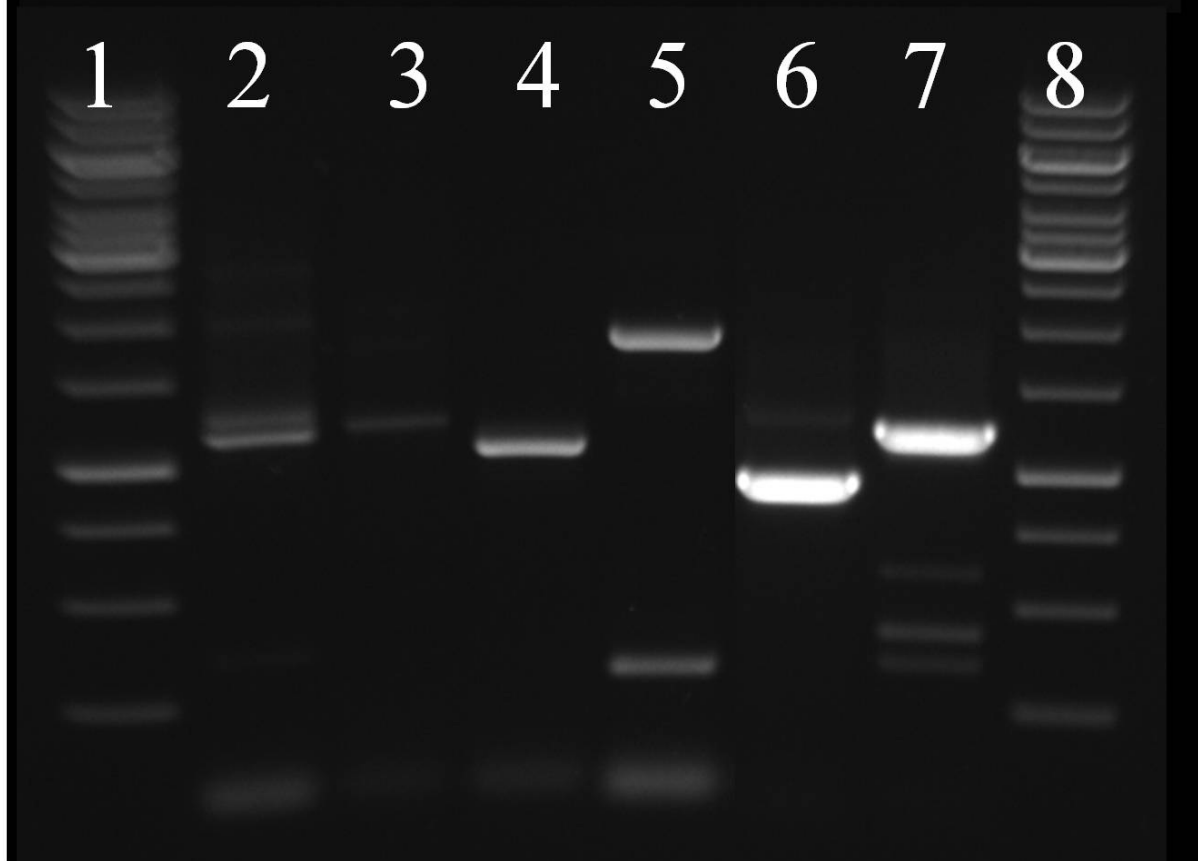

Supplement: Supplementary file 1 [file DataSheet1.ZIP › Supplemental Figure 3.pdf]
